# *Staphylococcus aureus* Small-Colony Variants from Airways of Adult Cystic Fibrosis Patients as Precursors of Adaptive Antibiotic-Resistant Mutations

**DOI:** 10.3390/antibiotics12061069

**Published:** 2023-06-17

**Authors:** Guillaume Millette, David Lalonde Séguin, Charles Isabelle, Suzanne Chamberland, Jean-François Lucier, Sébastien Rodrigue, André M. Cantin, François Malouin

**Affiliations:** 1 Département de Biologie, Faculté des Sciences, Université de Sherbrooke, Sherbrooke, QC J1K 2R1, Canada; guillaume.millette@usherbrooke.ca (G.M.); david.lalonde.seguin@usherbrooke.ca (D.L.S.); charles.isabelle@usherbrooke.ca (C.I.); suzanne.chamberland@usherbrooke.ca (S.C.); jean-francois.lucier@usherbrooke.ca (J.-F.L.); sebastien.rodrigue@usherbrooke.ca (S.R.); 2 Service de Pneumologie, Département de Médecine, Faculté de Médecine et des Sciences de la Santé, Université de Sherbrooke, Sherbrooke, QC J1H 5N4, Canada; andre.cantin@usherbrooke.ca

**Keywords:** *Staphylococcus aureus*, small-colony variant, cystic fibrosis, antibiotic resistance, resistance mutation, persister cells, MRSA

## Abstract

Prototypic *Staphylococcus aureus* and their small-colony variants (SCVs) are predominant in cystic fibrosis (CF), but the interdependence of these phenotypes is poorly understood. We characterized *S. aureus* isolates from adult CF patients over several years. Of 18 *S. aureus*-positive patients (58%), 13 (72%) were positive for SCVs. Characterization included genotyping, SCCmec types, auxotrophy, biofilm production, antibiotic susceptibilities and tolerance, and resistance acquisition rates. Whole-genome sequencing revealed that several patients were colonized with prototypical and SCV-related clones. Some clonal pairs showed acquisition of aminoglycoside resistance that was not explained by aminoglycoside-modifying enzymes, suggesting a mutation-based process. The characteristics of SCVs that could play a role in resistance acquisition were thus investigated further. For instance, SCV isolates produced more biofilm (*p* < 0.05) and showed a higher survival rate upon exposure to ciprofloxacin and vancomycin compared to their prototypic associated clones. SCVs also developed spontaneous rifampicin resistance mutations at a higher frequency. Accordingly, a laboratory-derived SCV (Δ*hemB*) acquired resistance to ciprofloxacin and gentamicin faster than its parent counterpart after serial passages in the presence of sub-inhibitory concentrations of antibiotics. These results suggest a role for SCVs in the establishment of persistent antibiotic-resistant clones in adult CF patients.

## 1. Introduction

Cystic fibrosis (CF) is a common life-shortening autosomal recessive disorder promoting chronic polymicrobial infections and bronchopulmonary inflammation that ultimately cause respiratory insufficiency. *Staphylococcus aureus* is one the most prevalent pathogens recovered from the lungs of CF patients [1,2]. Recent data show a noticeable increase in the prevalence of *S. aureus* among CF patients together with an increased life expectancy of adult patients [1,2]. The clinical impact of *S. aureus* in CF lung infections is not completely understood. Some studies noted a deleterious impact of *S. aureus* on airway inflammation, especially in children [3,4], whereas others reported no impact or even a better prognosis following *S. aureus* colonization [5,6,7]. However, it has been reported that *S. aureus* small-colony variants (SCVs) [8,9,10] and MRSA [11,12,13] worsen prognostics and increase mortality rate. A better understanding of the contribution of *S. aureus*, especially SCV and MRSA, to the health of adult CF patients is needed.

Subpopulations of SCVs are often found in the airways of CF patients [7,9,14]. In contrast to prototypical *S. aureus*, SCVs exhibit small, non-pigmented colonies less susceptible to aminoglycoside antibiotics due to their metabolic defect, which can often be restored by supplemental hemin, menadione, or thymidine [15,16]. SCVs express a set of virulence factors that favor host tissue colonization and intracellular persistence [14,17,18,19,20,21,22]. As a result, SCVs are associated with chronic infections, namely in the respiratory tract of patients with CF. 

Antibiotic prophylaxis and treatment regimens have been used in an attempt to reduce *S. aureus* chronic pulmonary infections [23,24]. While both resulted in fewer *S. aureus* infections, the positive impact on lung function was unclear [23,24]. However, it is clear that the increasing emergence of antibiotic-resistant bacteria in CF patients is a negative side-effect of such practices [25]. *S. aureus* manages to survive antibiotic treatments via different mechanisms, including biofilm production and the formation of SCVs [14,26]. While it is known that SCVs are inherently tolerant to many different antibiotics [16,27], it remains unclear whether they favor the acquisition of long-term resistance mechanisms that could be detrimental to CF patients in the long run. A recent study has shown that *E. coli* persister cells, a subpopulation of temporarily antibiotic-tolerant bacterial cells due to their slow growth [28], are able to resume growth after lethal stress, facilitating the development of clinical antibiotic resistance [29]. *S. aureus* SCVs, which are similar to persisters (i.e., slow growth and tolerance to antibiotics), may also facilitate the acquisition of antibiotic resistance. 

The impact of environmental factors on the incidence of *S. aureus* and SCVs is still not completely understood, but it is thought that exposure to some antibiotics may increase the presence of SCVs and persistent strains of *S. aureus*. Aminoglycoside therapy is associated with the formation of SCV auxotroph to hemin or menadione [20]. Trimethoprim-sulfamethoxazole treatments are linked to the emergence of thymidine-dependent SCV [30]. Exposure to fluoroquinolones induces the SOS response, which potentially increases the formation of SCVs [31]. Aside from antibiotic therapy, other factors can trigger the SCV phenotype: a pseudomonal quorum-sensing molecule, 4-hydroxy-2-heptylquinoline-*N*-oxide (HQNO) [32,33,34], the mammalian intracellular milieu [35], reactive aldehydes [36], permanent activation of the bacterial stringent response [37], CO_2_ auxotrophy [16], and oxidative stress [38]. As a result, new SCV-inducing factors and environmental conditions are uncovered, and such selective pressures could be more commonly found in infected patients than previously thought possible. 

Few studies have addressed the status of *S. aureus* and SCV colonization in CF patients and their contribution to the pathology (for a review, see [14,22]). Firstly, the aim of this work was to characterize *S. aureus* isolates from the respiratory tract of adult CF patients. We looked for the SCV phenotype, conducted genetic profiling, determined antibiotic susceptibility profiles, and measured biofilm production of *S. aureus* isolates. In addition, this study aimed to investigate how SCVs may contribute to the development of antibiotic resistance in the airways of CF patients, thus emphasizing the clinical importance of this phenotype.

## 2. Results

### 2.1. S. aureus Infections among CF Patients

For at least one visit, 18 (58%) of the 31 patients in this study tested positive for *S. aureus*. Except for patient 17, from whom only an SCV could be isolated, SCVs were isolated exclusively from patients colonized with prototypical *S. aureus*. SCVs were found among 13 (72%) of the 18 patients who had been colonized by *S. aureus*.

### 2.2. Genotype Profiles

From a total of 158 *S. aureus* isolates collected from the 18 patients who tested positive for *S. aureus* over the course of this study, 77 distinct *S. aureus* isolates were selected for further analyses. The individuality of these non-redundant 77 isolates was based on source (patient), colony morphology (prototype or SCV), type of auxotrophy if SCV, MLVA and *agr* typing, antibiotic susceptibility profile, and presence of the *mecA* gene (Table 1). All isolates were classified into 33 different profiles (A to GG) by MLVA. From these 77 distinct analyzed *S. aureus* isolates, a total of 26 SCV isolates were found. In some patients, prototypic *S. aureus* (large colonies; -L suffix in Table 1) and co-isolated SCVs (-S suffix in Table 1) had identical MLVA and *agr* types, indicative of genetic relatedness. From these distinct 77 analyzed *S. aureus* isolates, 12 MRSA isolates were found in this study and could be divided into three lineages based on MLVA and *agr* types (Table 1). All MRSA isolates had the SCCmec type II. 

**Figure 1:**
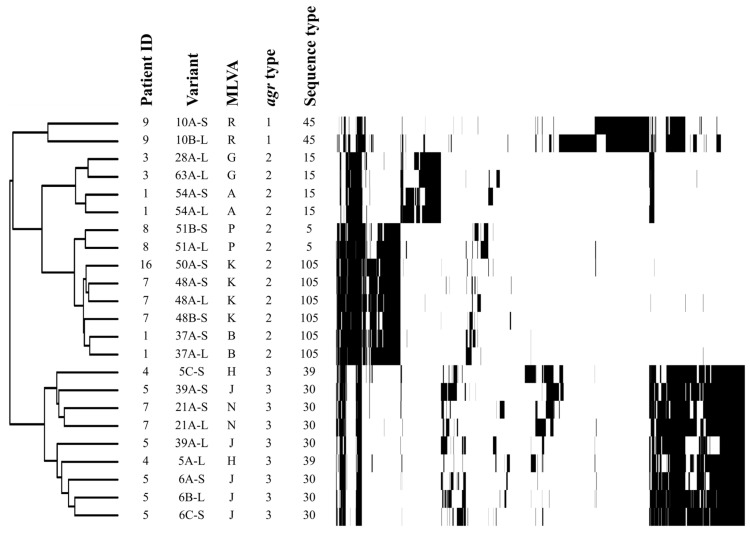
Phylogenetic tree of SCVs based upon SNP comparison. The phylogeny of the SCVs was assessed upon comparison of the presence/absence of high-impact SNPs (annotated by SNPeff) within each sequenced strain. A heatmap illustrating the dispersion of SNPs was also generated. Each vertical bar represents the presence of an SNP.

As mentioned above, MLVA and *agr* types suggested the presence of sequential clonal strains that persisted within the same patient for at least two visits (several months apart). Some of these clonal strains included prototypic–SCV pairs that were submitted to whole-genome sequencing. High-impact single-nucleotide polymorphisms (SNPs) were then used to create a phylogenetic tree to confirm the clonality of the sequential or prototypic–SCV pairs, as seen in Figure 1.

### 2.3. Auxotrophy of SCVs

Because their growth was enhanced by the presence of hemin and/or menadione, 12/26 (46%) of all SCVs appeared to be defective in electron transport. Three patients were colonized by a type of SCV for which abundant growth could only be restored by adding both menadione and hemin (Table 1). Only one thymidine-dependent SCV was found in a single patient. The majority of the SCVs retrieved from the patients (13/26 or 50%) were auxotroph for an unknown factor, meaning that their growth could not be complemented by either hemin, menadione, or thymidine.

### 2.4. Antibiotic Susceptibility Profiles

Table 1 shows the susceptibility profiles of the 77 distinct isolates to t10 different antibiotics. In the 18 patients who tested positive for *S. aureus*, 94% (17/18) were colonized by *S. aureus* isolates that were intermediate (I) or resistant (R) to at least one antibiotic (Table 1; see supplemental Table S1 for precise MIC values). Resistance to ciprofloxacin and erythromycin was found in 50% (9/18) and 61% (11/18) of these patients, respectively. Resistance to trimethoprim-sulfamethoxazole was found only in two patients (four distinct isolates) and was not exclusively associated with the presence of thymidine-dependent SCVs. Over the course of the study, 33% (6/18) of *S. aureus*-positive patients were colonized with MRSA (oxacillin MIC ≥ 64 µg/mL). All MRSA isolates were found to also be resistant to ciprofloxacin (MIC ≥ 64 µg/mL) and erythromycin (MIC ≥ 64 µg/mL). No resistance to rifampicin or vancomycin was found.

Since many SCVs were dependent on supplemental hemin or menadione or both for abundant growth, it was expected that the reduced proton motive force (PMF) of SCVs would reduce their susceptibility to aminoglycosides, which rely on an active PMF to cross the cell membrane barrier [39]. Indeed, a total of 23 SCVs out of 26 distinct SCV isolates (88%) showed an intermediate level of susceptibility (MIC of 8 µg/mL) to gentamicin or tobramycin, while 10 out of 26 (38%) were resistant (MIC > 32 µg/mL) to at least 1 aminoglycoside. Interestingly, in many cases, intermediate resistance or resistance to aminoglycosides seemed to gradually increase with time among isolates that were genetically related. Table 2 shows such cases found in eight different patients where the initial clone was susceptible to gentamicin or tobramycin and where other isolates from the same visit or from subsequent visits showed an increased resistance that could not be associated with acquisition of aminoglycoside-modifying enzymes. Most of the time, an SCV was found among those isolates, and this was also true for MRSA strains (Table 2).

### 2.5. Biofilm Production by S. aureus Isolates

**Figure 2:**
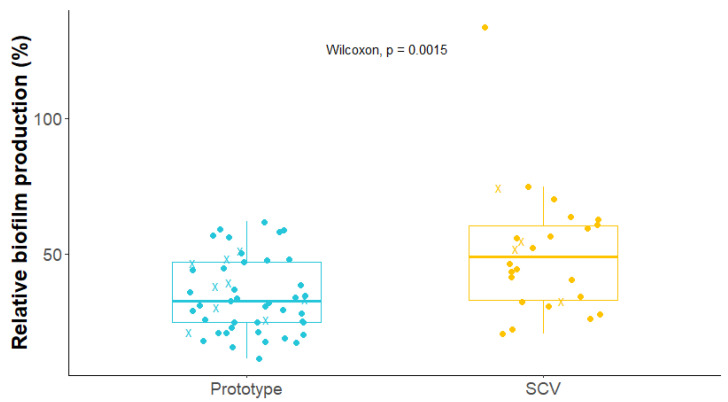
Biofilm production of prototypical and SCV *S. aureus* isolates collected from CF patients. The mean biofilm production for each isolate is indicated on the graph. Blue circles represent prototypic isolates and yellow circles represent SCVs. X symbols represent MRSA isolates. The horizontal bars represent the median relative biofilm production for each group, with the boxes showing the interquartile range and the whiskers indicating the minimum and maximum values (excluding outliers). The mean biofilm production (i.e., each dot) was calculated from at least three independent experiments in which each isolate’s biofilm formation was measured in four replicates. SCVs produced significantly more biofilm than prototypical *S. aureus* isolates (*p*-value = 0.0015; Wilcoxon test).

Because the presence of biofilm often impacts on the susceptibility of bacteria to antibiotics, the relative biofilm production capacity of prototypical and SCV isolates was determined. As shown in Figure 2, SCVs (n = 26) produced significantly more biofilm than prototypical (n = 51) S. aureus isolates (*p*-value = 0.0015; Wilcoxon test) with a median relative biofilm production that was 1.50-fold higher than that of the prototypical strains.

### 2.6. Kill Kinetics of Related SCV and Prototypic Isolates

**Figure 3:**
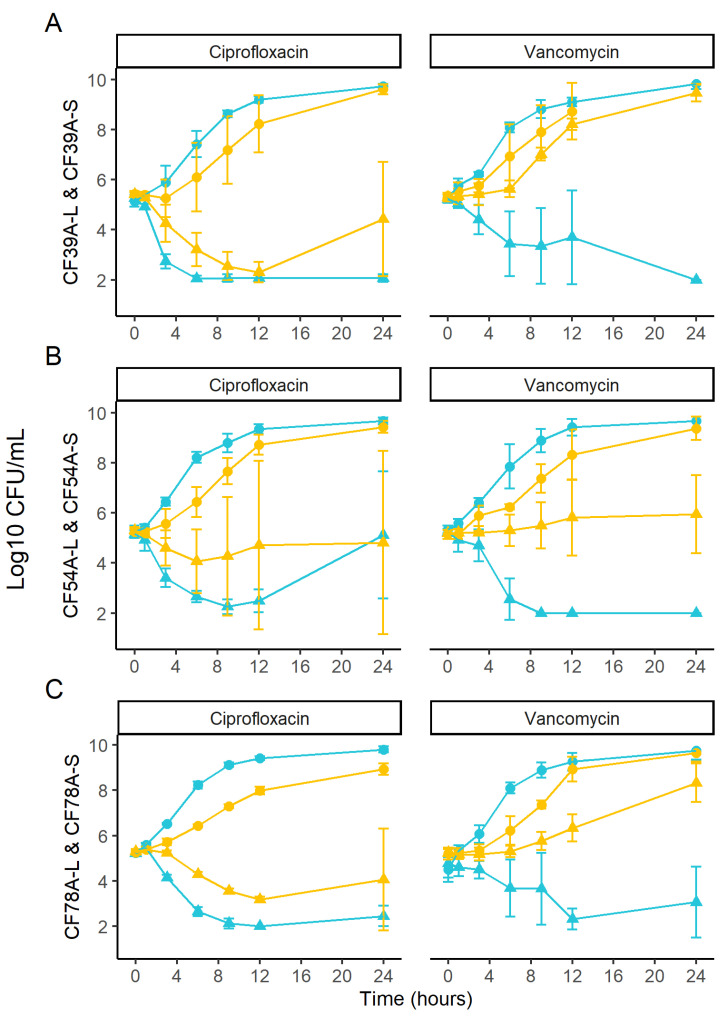
Ciprofloxacin and vancomycin kill kinetics in clinical prototype–SCV pairs. Blue symbols represent prototypic isolates (-L) and yellow symbols represent SCV (-S) isolates. Circles report the control condition (no antibiotic) and triangles report the kill kinetics with the indicated antibiotic. Standard deviations are also indicated. (**A**) represents CF39A-L and CF39A-S viable counts (CFU/mL); (**B**) represents CF54A-L and CF54A-S; (**C**) represents CF78A-L and CF78A-S. The detection limit was 2 log10 CFU/mL. Each condition was tested in triplicates and each symbol represents the mean.

We investigated whether antibiotics killed and/or inhibited growth of SCV and prototypical isolates to the same extent. To do so, we selected one related SCV–prototype pair from each of three different patients, resulting in a total of three pairs. The pairs used were CF54A, CF39A, and CF78A (-S and -L) from patients 1, 5, and 10, respectively. The related SCV and prototype isolates had identical MICs for ciprofloxacin and vancomycin, respectively (CF39A-L or -S: 2 and 1 µg/mL; CF54A-L or -S: 0.5 and 1 µg/mL; CF78A-L or -S: 0.25–0.5 and 1 µg/mL; MIC values can be found in Table S1). As expected, prototypes grew faster than SCVs in the control condition without antibiotic (Figure 3). On the other hand, SCVs better survived ciprofloxacin treatment, showing a much slower killing rate than that seen for prototypical isolates (Figure 3, left panels). Moreover, all three SCVs either maintained viability or showed growth in the presence of vancomycin compared to their related prototypical counterparts (Figure 3, right panels).

### 2.7. Higher Frequency of Rifampicin-Induced Resistant Mutants in SCVs

**Figure 4:**
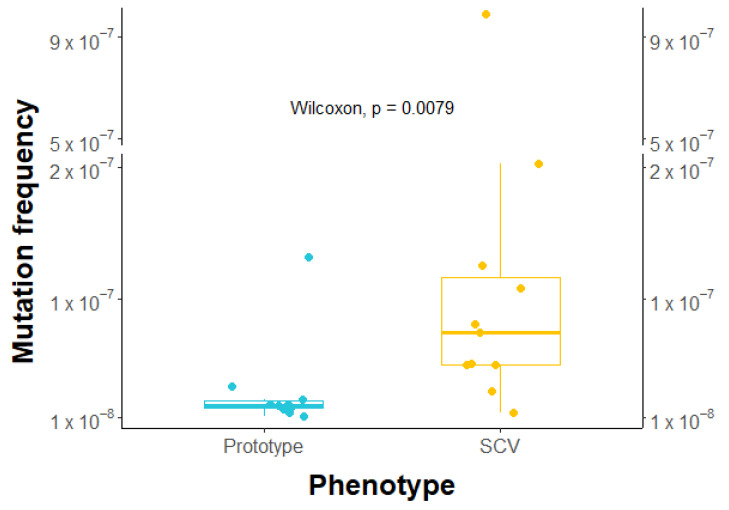
Comparison of rifampicin-induced mutation frequencies between prototypic isolates and SCVs. Ten prototypic isolates and eleven related SCVs were included in this analysis. Mutation frequency was determined by normalizing the number of resistant CFUs growing on the rifampicin plate by the initial inoculum. Blue circles represent prototypes and yellow circles are SCVs. The horizontal bars represent the mutation frequency median for each group, with the boxes showing the interquartile range, and the whiskers indicating the minimum and maximum values (excluding outliers). Results demonstrate that the mutation frequency leading to rifampicin resistance for SCVs is significantly higher than that measured for prototypic isolates (*p*-value = 0.0079; Wilcoxon test).

To assess whether SCVs tend to acquire beneficial mutations more easily than prototypic isolates under antibiotic pressure, we exposed 10 prototypes and 11 SCVs to rifampicin at a concentration equivalent to 10 times their MIC. Results showed that SCVs exhibited over three times higher mutant frequency than prototypes (median of 1.94 × 10^−8^ vs. 7.44 × 10^−8^, as shown in Figure 4), which was found to be statistically significant. These findings suggest that SCVs have a greater tendency to develop mutation-based antibiotic resistance than prototypes.

### 2.8. S. aureus Exposure to Sub-Inhibitory Concentrations of Gentamicin or Ciprofloxacin

**Figure 5:**
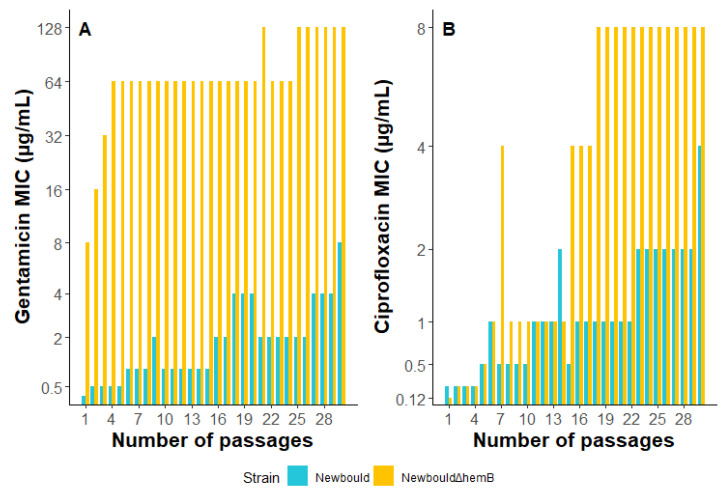
Selection of resistance using sequential passages on sub-inhibitory concentrations of antibiotics. *S. aureus* Newbould (blue bars) and the SCV NewbouldΔ*hemB* (yellow bars) were sequentially exposed to sub-inhibitory concentrations of gentamicin (**A**) or ciprofloxacin (**B**) for a total of 30 passages. The bars represent MIC values (the highest value is >128 and >8 µg/mL, respectively, for gentamicin and ciprofloxacin).

*S. aureus* Newbould and NewbouldΔ*hemB* were sequentially exposed to sub-inhibitory concentrations of gentamicin and ciprofloxacin for a total of 30 passages (Figure 5). A NewbouldΔ*hemB*-resistant population (MIC > 16 µg/mL) emerged after only 2 passages when exposed to gentamicin and even reached an MIC of 128 µg/mL after 30 passages, whereas the bacterial population derived from the prototypical strain Newbould only achieved an intermediate level of resistance (MIC of 8 µg/mL) after all the passages. Furthermore, both strains produced ciprofloxacin-resistant populations (MIC > 4 µg/mL) but Newbould achieved resistance after 30 passages, whereas NewbouldΔ*hemB* only required 15 passages to achieve and maintain resistance. These results suggest that in the presence of antibiotics, SCVs may develop higher levels of resistance, and more rapidly, than prototypical *S. aureus*.

## 3. Discussion

*S. aureus* is one of the most frequently isolated pathogen in the CF respiratory tract [1,7]. Although its prevalence is particularly high in the earlier years of life, it is still common for adults to be infected. This study reports the prevalence and the characteristics of *S. aureus* in an adult CF patient population. In agreement with previous studies [40,41], genotyping results demonstrated the important diversity of *S. aureus* isolates that can colonize the airways of CF patients (within and across patients). The co-isolation of *S. aureus* normal-growth (prototypic) and SCV phenotypes with identical genotype profiles may reflect the phenotype-switching phenomenon previously observed in other studies of CF patients [21,42]. On the other hand, the detection of isolates with similar genotypic profiles among different patients, such as some of the MRSA isolates detected in this study, may reflect nosocomial transmission from non-CF patients or cross-contamination between patients frequenting the same CF clinic. Indeed, CF50A-S (patient 16), CF48A-S, and CF48A-L (patient 7) are all MRSA and were closely related (Figure 1). According to their sequence type, they all belong to the clonal complex 5, which includes hospital-associated MRSA USA100 [43]. While it has frequently been reported for *P. aeruginosa* [44,45], transmission of *S. aureus* between patients is not as frequent [46,47]. In our present study, all isolated MRSA showed identical MLVA profile, *agr* type, and SCC*mec* type.

In addition to its genetic diversity, *S. aureus* isolates showed multiple antibiotic resistance profiles (Table 1). Of interest, several aminoglycoside-intermediate or resistant strains of *S. aureus* for which resistance was not explained by the acquisition of common AMEs (Table 2) were identified in this study. As suggested before, such phenotypes may reflect the adaptability of *S. aureus* in stressful environments such as the respiratory tract of CF patients [48]. 

In this study, SCVs were superior biofilm producers compared to prototypical isolates (Figure 2). Biofilms often contain subpopulations of persister cells. It has previously been suggested that metabolically inactive persister cells tolerate high antibiotic exposure, even for concentrations above the determined MIC of the strain [49], and that this favors the emergence of antibiotic resistance [29]. Since SCVs both produce large amounts of biofilm and already share many similarities with persister cells (e.g., slow growth), we investigated whether antibiotics killed SCV and prototypic isolates in the same way (Figure 3). In a time-kill assay comparing prototypic and SCV isolates showing identical antibiotic MICs, SCVs demonstrated greater survival than their genetically related prototypic counterparts in the presence of ciprofloxacin or vancomycin. Among the different features allowing vancomycin resistance, two that could apply to SCVs have previously been described: cell wall thickening and a reduced growth rate [50]. While the SCVs’ reduced growth rate is self-explanatory, *sigB* overexpression, a typical trait of the SCVs [33,51], is associated with cell wall thickening [52] and has also been described in vancomycin-resistant *S. aureus* strains [53]. As for the SCVs’ increased tolerance to ciprofloxacin, a possible explanation is that the SCV is less susceptible to the reactive oxygen species (ROS) induced by ciprofloxacin [38]. A longer exposure to ciprofloxacin would then be required to obtain a lethal level of ROS in SCVs. 

After testing the kill kinetics, it was assessed whether SCVs had a higher mutation frequency compared to prototypic isolates (Figure 4), which is a characteristic of persister cells [29]. Related SCV and prototypical clinical strains were exposed to rifampicin at concentrations exceeding their MIC, and resistant CFUs were counted and normalized to the initial inoculum. SCVs were found to be over 3 times more likely to develop resistance to rifampicin, and the range of mutation frequencies observed for SCVs was wider than that measured for prototypic isolates. Further studies could explore the cause of resistance in the mutants by whole-genome sequencing and what drives some SCVs to acquire resistance more easily. Overall, this increased mutation rate could represent another factor facilitating antibiotic resistance development over time. 

The effect of sub-MIC concentrations of gentamicin and ciprofloxacin on the emergence of resistance in SCVs versus prototypic isolates was also investigated (Figure 5). For both antibiotics, SCVs developed higher resistance levels earlier than prototypic isolates. One possible explanation is the greater tolerance of SCVs to killing by antibiotics, which allows a larger proportion of the population to survive (Figure 3). With this, combined with the higher frequency of mutations shown in Figure 4, SCVs would then be more prone to developing beneficial mutations for resistance in fewer passages (Figure 5). However, the precise cause of resistance was not determined. Whole-genome sequencing of the isolates at different passages could provide valuable information about intermediate-step mutations and ultimate full resistance mutations occurring in such experiments. Furthermore, additional passages in antibiotic-free media could be performed to verify if the resistance of both SCV and prototype is maintained. Further research is needed to better understand the specific mechanisms of resistance to the antibiotics. 

New studies investigating the impact of SCVs on patient health should include robust and extensive means of detection of SCVs, considering unknown auxotrophies. Most of the SCVs retrieved in this study were auxotroph for an unknown factor. As stated earlier, the SCV phenotype may be triggered in several forms [8,17,31,32,33,34,35,36,37,38,54,55] and many may remain undiscovered in the clinical laboratory. Notably, while we have tested for the most common auxotrophies (hemin, menadione, and thymidine), we did not test reversion of the SCV phenotype by CO_2_ or fatty acid complementation, which also have previously been described to be responsible for the SCV phenotype [55,56,57,58]. Among our collection of isolates, we found a menadione-dependent SCV (patient 7, CF21A-S) that could be complemented for normal growth and pigmentation by its co-prototypical strain (CF21A-L, Figure S1). A similar phenomenon was previously observed with thymidine-dependent SCVs [59], but never with SCVs of other auxotrophies. Even if factors providing normal growth to SCVs remain to be determined, inter-strain complementation may importantly contribute to the course of an infection. Indeed, the capacity of SCVs to grow normally in the presence of their prototypical counterparts may increase their fitness in *S. aureus*-favorable environments and inversely allow a rapid return to the SCV persistent phenotype in harsh environments [42].

The use of MLVA and *agr* typing (Table 1), and to some extent whole-genome sequencing (Figure 1), allowed the identification of sequential clonal strains and prototypic–SCV pairs persisting in patients. Their presence certainly represents the capacity of *S. aureus* to colonize adult CF airways successfully for months. Since we found SCVs to withstand antibiotics better and to establish resistance faster than prototypic isolates, we propose that SCVs can facilitate faster acquisition of antibiotic resistance in subsequent sequential isolates. Whole-genome sequence analyses should help to identify mutations inducing the SCV phenotype and/or antibiotic resistance. For example, CF5C-S and CF48B-S, two SCVs whose auxotrophies were not supplemented by hemin, menadione, or thymidine, were mutated in the *cysG* and *menA* genes, respectively, which encode enzymes involved in either heme or menaquinone biosynthesis (Table S2). Further analysis is required to determine if and how these mutations are responsible for the SCV phenotype, but they most likely affect the respiratory chain. Furthermore, a preliminary review of the SNPs that were used to demonstrate clonality of related prototypic and SCV pairs (Figure 1) identified some SNPs impacting DNA repair among different SCVs, e.g., dUTP pyrophosphatase, deoxyribose-phosphate aldolase, and recombinase. As previously described, strains defective for DNA repair and maintenance are often hypermutators with an increased mutation rate that favors the emergence of antibiotic resistance [60]. SCVs displaying such SNPs could facilitate acquisition of full antibiotic resistance in either the SCV or prototypical phenotypes since these can be reversible. As an example, SNPs were more closely examined in three sequential isolates showing important changes in their antibiotic susceptibility profiles (Table 2 and Table S3; mutations in Table S3 were confirmed by Sanger sequencing). When isolates CF63A-S and CF63A-L were compared to their sequential clone precursor, CF28A-L, all three isolates showed a frameshift SNP in the *ksgA* gene. This gene’s functions are to catalyze proper processing of rRNA during ribosome biogenesis and to favor translation fidelity [61]. It also has a DNA glycosylase-apurinic/apyrimidinic site lyase activity, allowing repair of mismatched DNA strands [62]. In previously published articles, it has been shown that *ksgA* mutations not only conferred modest resistance to kasugamycin, an aminoglycoside, but also favored higher rates of high-level-resistance mutants [63]. These results suggest that the inactivation of *ksgA* might facilitate the acquisition of mutations for antibiotic resistance, which would be coherent with our observations. Furthermore, CF63A-S and CF63A-L, but not the precursor CF28A-L, share a moderate-impact SNP (amino-acid substitution) in gene *lacD* (Table S3). Interestingly, a study investigating amicoumacin A resistance in *S. aureus* identified both *ksgA* and *lacD* as mutated genes responsible for an increase in resistance [64]. However, contradictory results were obtained for other aminoglycosides (e.g., kanamycin, gentamicin, and tobramycin), as a *ksgA* mutant was found to be more susceptible to these [65]. Furthermore, at this time, the role of LacD in *S. aureus* antibiotic resistance seems farfetched, but the closely related Class I aldolase LacD.1 in *Streptococcus pyogenes* was proposed to influence the transcription of virulence genes through a direct and specific association with the transcription regulator RopB (Rgg) [66,67,68]. Further experiments should be conducted to evaluate the role of *ksgA* and *lacD* in the emergence of antibiotic resistance.

In conclusion, while SCVs have previously been identified as contributing to the persistence of infection in the CF airway [14,21], SCVs can potentially represent yet another challenge since they can acquire mutational antibiotic resistance more easily than prototypical strains (Figure 5). Due to their high biofilm production (Figure 2), ability to tolerate antibiotic action (Figure 3), and increased beneficial mutation rate (Figure 4), SCVs could accumulate useful mutations under selective pressure (e.g., antibiotic therapy) and then revert to a normal-growth and more virulent phenotype with a now reduced susceptibility to antibiotics. This study also identified associated prototypic–SCV MRSA pairs in some patients, which is further cause for concern. Hence, the phenotypic switch from SCVs to prototypic *S. aureus* and vice versa apparently occurring in patients may represent properties that favor the establishment and persistence of *S. aureus* in adult CF patients and could also reduce antibiotic susceptibility over time. Since modulators of the CFTR function have been shown to reduce CF lung colonization by MRSA [69], it remains to be seen if such novel therapies could also prevent the emergence of SCVs and break this vicious circle. 

## 4. Materials and Methods

### 4.1. Reference Strains and Growth Conditions

The *S. aureus* reference strains used in this study were ATCC 29213, Newbould (ATCC 29740), and its stable SCV derivative NewbouldΔ*hemB* [70]. ATCC 29213 was used for quality control in antibiotic susceptibility tests with clinical isolates, while Newbould and NewbouldΔ*hemB* were used for serial passage on sub-inhibitory concentrations of gentamicin and ciprofloxacin. Unless otherwise stated, TSA and TSB (BD, Mississauga, ON, Canada) were generally used as growth media.

### 4.2. Patients and Specimens

A total of 31 adult patients (12 males and 19 females) regularly treated at the Cystic Fibrosis outpatient clinic of the Centre Hospitalier Universitaire de Sherbrooke (CHUS, Sherbrooke, QC, Canada) were enrolled in the study from 2008 to 2018. The age of patients ranged from 18 to 41 years old (mean of 25). Specimens were obtained from sputum samples or deep throat swabs when no bronchial secretions were produced. For each patient, specimens from a routine visit or hospitalization (exacerbations) were collected and analyzed. Every patient visit was separated by three to eight months. This study was approved by the ethics review board of the Centre Intégré Universitaire de Santé et de Services Sociaux de l’Estrie (CIUSSS)—CHUS, and prior informed consent was obtained from all subjects recruited in this study (ethics protocol #2008-82, 06-158).

### 4.3. Microbiology of Clinical Samples

For all specimens, detection of *S. aureus* was performed using routine clinical laboratory methods. To further confirm the presence of prototypical *S. aureus* and to facilitate detection of SCVs, a portion of each sample was also treated as follows. For sputum samples, 100 µLof dithiothreitol (100 mg/mL) was added to 100 mg of sputum [71,72]. From this solution, 100 µL was then added to 10 mL of “NaCl broth” that consisted of 7.5 g of tryptone, 1.25 g of neutralized liver digest, 2.5 g of yeast extract, 37.5 g of NaCl, 5 g of (D-) mannitol, 2 g of glucose, and 0.5 g of thymidine in a final volume of 500 mL. For deep throat specimens, swabs were directly used to inoculate 10 mL of NaCl broth. Cultures were aerobically incubated at 35 °C with shaking (225 rpm) for 48 h. Aliquots were then spread on two different media. Mannitol-salt agar (MSA) was used for detection of prototypical *S. aureus,* while brain-heart infusion agar (BHIA) supplemented with 10 µg/mL of nalidixic acid, 10 µg/mL of polymyxin B, and 2 µg/mL of gentamicin (BHIA-SCV) was used to suppress *Pseudomonas* sp. and to support the growth of SCVs [71,73,74,75].

From each sample, a maximum of 8 prototypical *S. aureus* and SCV colonies were isolated from MSA or BHIA-SCV. Very small and non-pigmented colonies were considered to be SCVs. All prototypical colonies and SCVs were confirmed to be *S. aureus* by PCR detection of the *femA* gene [76] and of the 668 pb product amplified from the *nuc* gene by using the following primer sequences: *nuc* FWD 5′-GGCATCTAGAGCTAAGTCGTGGCATATGTATGGC-3′ and *nuc* REV 5′-CCGCACTAGTCCTTGACCTGAATCAGCGTTG-3′. The *mecA* gene coding for the low-affinity penicillin-binding protein responsible for methicillin resistance in *S. aureus* [77] was also identified by PCR. All isolates were kept frozen at −80 °C. The *S. aureus* clinical isolates obtained are described in Table 1.

### 4.4. Genotyping and Selection of Representative Isolates

Multiple-locus variable-number of tandem repeat analysis (MLVA) was use to address the genetic profiles of confirmed *S. aureus* colonies [78]. The MLVA is based on five loci: *sdr*, *clfA*, *clfB*, *ssp*, and *spa*. Band profiles were both visually evaluated and analyzed by using Quantity One software (version 4.6.3 BioRad, Munich, Germany). Any two MLVA patterns differing by one or more bands were considered distinct types. Accessory gene regulator (*agr*) typing was carried out by group-specific multiplex PCR amplification, as described previously [79]. For each patient, a selection of representative isolates was made based on MLVA and *agr* profiles, the presence or absence of *mecA* (and SCC*mec* types—see below), the normal or SCV phenotype, and the various auxotrophies for SCVs. Clones were defined as strains with the same MLVA and *agr* profile, while sequential clonal strains were defined as clones isolated from distinct visits of the same patients. 

### 4.5. Genome Sequencing and Annotation

To confirm the clonality of clinical isolates previously determined by MLVA typing, 23 sequential clonal strains taken from lineages that acquired significant antibiotic resistance and included SCV isolates were further analyzed using Illumina sequencing. DNA was first extracted using the GenElute bacterial genomic DNA kit (Sigma–Aldrich, Oakville, ON, Canada). Purifications were made on double solid phase reversible immobilization (SPRI) with carboxyl-coated magnetic beads with an adjusted ratio of 0.7, to obtain fragments longer than 300 bp. Using the QIAseq FX DNA library kit (Qiagen, Toronto, ON, Canada), adapters were ligated, then the libraries were amplified by PCR and validated using a 2100 Bioanalyzer (Agilent Technologies, Mississauga, ON, Canada). The Quant-iT PicoGreen dsDNA assay kit (Invitrogen, Burlington, ON, Canada) was used to quantify gDNA before pooling libraries. Library pool sequencing was performed on an Illumina HiSeq 2000 sequencing system at the *Centre d’innovation* Genome Quebec platform to generate 125 bp paired-ends reads. Sequenced data quality was verified using the FastQC control tool v0.11.4. Briefly, sequencing reads where trimmed using trimmomatic v0.32 with the following parameters: ILLUMINACLIP:new_illumina_adaptors.fa:2:30:15 TRAILING:30 MINLEN:50. Afterwards, reads were aligned with bwa mem v0.7.10 using reference genome from NCBI Staphylococcus aureus subsp. aureus str. Newman NC_009641.1 and sorted by coordinates using picard v1.123. GATK v3.3.0 RealignerTargetCreator and IndelRealigner steps were run on aligned reads. Picard v1.123 MarkDuplicates is executed to locate and tag duplicate reads in a BAM along with metrics file. Next, the VCF file was generated using GATK v3.3.0 HaplotypeCaller module on previously generated bam using these parameters: --emitRefConfidence GVCF --variant_index_type LINEAR --variant_index_parameter 128000 -dt none -nct 1. Finally, the VCF file was annotated using the snpEff v3.6 using database Staphylococcus_aureus_subsp_aureus_str_newman and the following parameters: -noStats -ud 0. 

Finally, a phylogenetic tree was created by comparing the SNP difference between the sequenced strains. All VCF files from every strain were imported to a custom Sqlite database and queried for high-impact snps. These high-impact snps were then clustered using R 4.1.2 with hclust, using the complete method where distance was computed using the dist function and the manhattan method. Cran package gplots was used to generate heatmap. Sequences of the 23 sequential clonal strains were submitted on PubMLST (https://pubmlst.org/saureus/ accessed on 15 May 2023) to determine their sequence type, using 7 house-keeping genes (*arcC, aroE, glpF, gmk, pta, tpi, and yqi*).

### 4.6. Minimal Inhibitory Concentrations (MICs)

Antibiotic susceptibility profiles were evaluated by a broth microdilution method as recommended by the Clinical and Laboratory Standards Institute [80] with some modifications. Trimethoprim-sulfamethoxazole (T-S at a ratio of 1:19) was evaluated using Cation Adjusted Mueller-Hinton Broth (CAMHB). Tobramycin (TOB), gentamicin (GEN), ciprofloxacin (CIP), erythromycin (ERY), tetracycline (TET), rifampicin (RIF), and vancomycin (VAN) were tested using BHI to allow substantial growth of all *S. aureus* isolates including SCVs. Resistance for clindamycin (CLI) was tested for all isolates of the first visit (Table 1, V1) and erythromycin-resistant isolates were further tested for inducible resistance to CLI by the ERY-CLI D-test [80]. For thymidine-dependent SCVs, which showed reversion in BHI, MICs for GEN, TOB, and T-S were performed using CAMHB. SCVs with unknown auxotrophy were tested using both BHI and CAMHB. Oxacillin (OXA) was tested in CAMHB supplemented with 2% NaCl. All tested antibiotics were purchased from Sigma-Aldrich (Oakville, ON, Canada).

### 4.7. Staphylococcal Cassette Chromosome mec (SCCmec) Typing

SCC*mec* types were detected using a multiplex PCR assay described previously [81] on all oxacillin-resistant isolates (≥4 µg/mL; referred as MRSA in the text). 

### 4.8. Aminoglycoside Resistance Determinants

The aminoglycoside-resistant isolates in this study were screened by PCR for the presence of genes encoding aminoglycoside-modifying enzymes (AMEs) as described in Mitchell et al. [82].

### 4.9. Auxotrophy of SCVs

Auxotrophy of SCVs for hemin, thymidine, and menadione was determined using an agar diffusion method. Directly taken from the frozen culture stock and following overnight growth on TSA, 3 to 4 SCV colonies were pooled and evenly spread onto new TSA plates. Wells were punched into the TSA and supplements were added as follows: hemin or menadione each at 10 µg/well; a mixture of both hemin (10 µg) and menadione (10 µg) in the same well; and thymidine at 100 µg/well. Auxotrophy was detected by abundant growth of normal-sized colonies around wells containing specific supplement or combination of supplements after 18 h of incubation at 35 °C. The assay was carried out in duplicate.

### 4.10. Biofilm Production

The extent of biofilm production was measured by spectrophotometry after growth in 96-well microtiter plates using crystal violet staining, as previously described [17]. The results were collected from at least three independent experiments in which each strain’s biofilm formation was measured in four replicates. The mean biofilm production of each individual strain was expressed relatively to the mean biofilm production of the calibrator strain *S. epidermidis* ATCC 35984 (100%) measured at the same time in each 96-well plate and all experiments. 

### 4.11. Kill Kinetics

Antibiotic efficacy against SCVs and prototypical isolates was studied in time-kill experiments, as described in Boulanger et al. [83]. Pairs of genetically related SCVs and prototypical isolates co-isolated in patients were selected for this in vitro study. Bacteria were inoculated at 10^5^–10^6^ CFU/mL. BHI was used for SCVs and CAMHB was used for prototypical isolates; these conditions were chosen so that their growth density could be comparable after 24 h. The cultures were incubated for 24 h at 35 °C with shaking (225 rpm). Samples were taken prior to the addition of antibiotics (T = 0) and at several time points during the 24 h incubation period. Samples were serially diluted and plated on TSA to determine viable bacterial counts. TSA plates were incubated 24 h at 35 °C for prototypical isolates, while for SCVs the incubation was prolonged to 48 h at 35 °C to allow detection of growth. Ciprofloxacin and vancomycin were used at the MIC of each pair of strains. Data were collected from at least three independent assays.

### 4.12. Selection of Rifampicin-Resistant Mutants in Prototypic and SCV Backgrounds

Pairs of genetically related SCVs and prototypical isolates co-isolated in patients were selected for this experiment. The selected SCVs (-S) and prototypic (-L) isolates were: Patient 1, CF37A-S, CF54A-S, CF37A-L, CF54A-L; Patient 3, CF63A-S, CF28A-L, CF63A-L; Patient 5, CF6A-S, CF6C-S, CF39A-S, CF6B-L, CF39A-L; Patient 7, CF21A-S, CF48A-S, CF48B-S, CF21A-L, CF48A-L; Patient 8, CF51B-S, CF51A-L; Patient 16, CF50A-S, CF50A-L. Bacteria were suspended in PBS, and approximately 3 × 10^8^ CFU were plated in triplicates on TSA plates containing 0.3 µg/mL of rifampicin (i.e., ~10–30 × MIC). Plates were incubated 24 h at 35 °C for prototypical isolates, while for SCVs the incubation was prolonged to 48 h at 35 °C to allow detection of growth. CFUs were normalized with the exact initial inoculum on plates. Data were collected from three independent assays. 

### 4.13. Selection of Resistance Using Sub-Inhibitory Concentrations of Antibiotics

Sequential passages into broth containing sub-inhibitory concentrations of gentamicin or ciprofloxacin were used to compare the rate of emergence of resistant mutants from the prototypical *S. aureus* strain Newbould and its SCV derivative NewbouldΔ*hemB*. In 96-well plates, gentamicin and ciprofloxacin were serially diluted 2-fold in TSB, yielding concentrations ranging from 128 to 0.25 µg/mL and 8 to 0.015 µg/mL, respectively. A bacterial suspension of 10^5^ to 10^6^ CFU/mL was then inoculated into the wells. Following a 24 h or 48 h incubation at 35 °C for Newbould and NewbouldΔ*hemB*, respectively, MICs were determined, and cultures growing one dilution below the MIC breakpoint were used to inoculate a new series of plates containing antibiotics. A total of 30 passages were completed for each strain and MICs were determined at every passage. 

## Figures and Tables

**Table 1:** Clonal diversity, antibiotic susceptibilities, and phenotypic characteristics of 77 *S. aureus* isolates from 18 adult CF patients.

Patient ID	Visit	First Isolate	Variant	MLVA	*agr*	*mecA*	Antibiotic Susceptibility ^a^										Aux ^b^
							OXA	TOB	GEN	T-S	CIP	ERY	CLI	TET	VAN	RIF	
1	V1	CF1A-L		A	2		S	S	S	S	S	S	S	S	S	S	
	V1		CF1C-S	A	2		S	I	I	S	S	S	S	S	S	S	H
	V1		CF1D-S	A	2		nt	I	S	nt	S	S	R	S	S	S	U
	V2		CF37B-S	A	2		S	I	I	S	R	R	nt	S	S	S	U
	V3		CF54A-L *	A	2		S	S	S	S	S	S	nt	S	S	S	
	V4		CF54A-S *	A	2		S	R	R	S	S	S	nt	S	S	S	U
	V2	CF37A-L *		B	2	+	R	S	S	S	R	R	nt	S	S	S	
	V2		CF37A-S *	B	2	+	R	S	S	S	R	R	nt	S	S	S	U
2	V1	CF2A-L		C	1		S	S	S	S	S	S	S	S	S	S	
	V3		CF62B-L	C	1		S	S	S	S	S	S	nt	S	S	S	
	V1	CF2C-L		D	3		S	S	S	S	S	S	S	S	S	S	
	V1		CF2B-S	D	3		S	I	S	S	S	S	S	S	S	S	H
	V3		CF62A-L	D	3		S	S	S	S	S	S	nt	S	S	S	
	V2	CF34A-L		E	1		S	S	S	S	S	S	nt	S	S	S	
	V2	CF34B-L		F	3		S	S	S	S	S	S	nt	S	S	S	
3	V1	CF4B-L		A	2		S	S	S	S	S	S	S	S	S	S	
	V1		CF4B-S	A	2		S	I	I	S	S	S	S	S	S	S	M
	V2		CF28B-L	A	2		S	S	S	S	S	S	nt	S	S	S	
	V2	CF28A-L *		G	2		S	S	S	S	S	R	nt	S	S	S	
	V3		CF63A-S	G	2		S	R	S	S	S	R	nt	S	S	S	U
	V3		CF63A-L *	G	2		S	R	I	S	R	R	nt	S	S	S	
4	V1	CF5A-L *		H	3		S	S	S	S	S	S	S	S	S	S	
	V1		CF5C-S *	H	3		nt	I	I	S	S	S	S	S	S	S	U
	V1	CF5E-S		I	3		S	I	I	S	S	S	S	S	S	S	H+M
5	V1	CF6B-L *		J	3		S	R	R	S	S	S	S	S	S	S	
	V1		CF6A-S *	J	3		S	R	R	R	S	S	S	S	S	S	T
	V1		CF6C-S *	J	3		S	R	R	S	S	S	S	S	S	S	H+M
	V2		CF39A-L *	J	3		S	R	R	R	S	S	nt	S	S	S	
	V2		CF39A-S *	J	3		S	R	R	R	S	I	nt	S	S	S	U
6	V1	CF7A-L		K	2	+	R	R	S	S	R	R	R	S	S	S	
	V1		CF7D-L	K	2		S	S	S	S	R	R	R	S	S	S	
	V2		CF27A-L	K	2	+	R	R	S	S	R	R	nt	S	S	S	
	V3	CF49A-L		L	3		S	S	S	S	S	S	nt	S	S	S	
	V3	CF49A-S		M	3		S	I	S	S	S	S	nt	S	S	S	H
	V3		CF49B-S	M	3		S	I	S	S	S	S	nt	S	S	S	U
7	V1	CF8A-L		N	3		S	S	S	S	S	S	S	S	S	S	
	V2		CF21A-L *	N	3		S	S	S	S	S	S	nt	S	S	S	
	V2		CF21A-S *	N	3		S	S	S	S	S	S	nt	S	S	S	M
	V1	CF8C-L		O	3		S	S	S	S	S	S	S	S	S	S	
	V1		CF21D-L	O	3		S	S	S	S	S	S	nt	S	S	S	
	V1	CF8D-L		P	3		S	S	S	S	S	S	S	S	S	S	
	V3	CF48A-L *		K	2	+	R	R	S	S	R	R	nt	S	S	S	
	V3		CF48A-S *	K	2	+	R	R	S	S	R	R	nt	S	S	S	U
	V3		CF48B-S *	K	2		S	R	I	S	R	R	nt	S	I	S	U
8	V1	CF9A-L		K	2	+	R	R	S	S	R	R	R ^c^	S	S	S	
	V1		CF9F-L	K	2		S	R	R	S	R	R	I	S	S	S	
	V2	CF51A-L *		P	2		S	S	S	S	R	R	nt	S	S	S	
	V2		CF51B-S *	P	2		S	I	I	S	R	R	nt	S	I	S	U
	V3		CF75B-L	P	2		S	S	S	S	R	R	nt	S	S	S	
	V3	CF75A-L		Q	1		S	S	S	S	R	S	nt	S	S	S	
9	V1	CF10B-L *		R	1		S	S	S	S	S	R	R	S	S	S	
	V1		CF10A-S *	R	1		S	R	R	S	S	R	R	S	S	S	H + M
10	V1	CF18A-L		S	2		S	S	S	S	S	R	R ^c^	S	S	S	
	V2		CF33A-L	S	2		S	S	S	S	I	R	nt	S	S	S	
	V3		CF78A-L	S	2		S	I	S	S	S	S	nt	S	S	S	
	V3		CF78A-S	S	2		S	I	S	S	S	S	nt	S	S	S	M
	V3		CF78B-S	S	2		S	I	S	S	S	S	nt	S	S	S	H
	V1	CF18E-L		T	1		S	S	S	S	S	S	S	S	S	S	
11	V1	CF19A-L		T	1		S	S	S	S	R	S	S	S	S	S	
	V1	CF19B-L		U	1		S	S	S	S	R	S	S	S	S	S	
	V1	CF19A-S		V	1		S	R	R	S	S	S	S	S	S	S	U
	V2	CF41A-L		W	1		S	S	S	S	R	R	nt	S	S	S	
	V2		CF41A-S	W	1		S	I	S	S	R	S	nt	S	S	S	U
	V3	CF58A-L		X	1		S	R	R	R	S	S	nt	S	S	S	
	V3	CF58B-L		Y	1		S	I	S	S	R	S	nt	S	S	S	
12	V1	CF22A-L		Z	1		S	S	S	S	S	S	S	S	S	S	
13	V1	CF29A-L		AA	1		S	I	I	S	S	S	R	S	S	S	
14	V1	CF35A-L		K	2	+	R	S	S	S	R	R	R ^c^	S	S	S	
	V2		CF77A-L	K	2	+	R	S	S	S	R	R	nt	S	S	S	
15	V1	CF43A-L		BB	3		S	S	S	S	R	R	R	S	S	S	
	V1	CF43B-L		CC	3		S	S	S	S	R	R	R	S	S	S	
16	V1	CF50A-L		K	2	+	R	R	S	S	R	R	S	S	S	S	
			CF50A-S *	K	2	+	R	R	I	S	R	R	S	S	S	S	H
	V2	CF111A-L		DD	2	+	R	R	S	S	R	R	nt	S	S	S	
17	V2	CF86A-S		EE	2		S	S	S	S	S	S	nt	R	S	S	H
18	V1	CF81A-L		FF	3		S	S	S	S	S	S	S	S	S	S	
	V2	CF91A-L		GG	1		S	S	S	S	S	S	nt	S	I	S	

^a^ Ciprofloxacin (CIP), clindamycin (CLI), erythromycin (ERY), gentamicin (GEN), oxacillin (OXA), rifampicin (RIF), tetracycline (TET), tobramycin (TOB), trimethoprim-sulfamethoxazole (T-S), and vancomycin (VAN). Not tested (nt), susceptible (S), intermediate (I), and resistant (R). ^b^ Aux, auxotrophy for the normal-growth phenotype: hemin (H), menadione (M), thymidine (T), unknown (U). ^c^ These isolates are presumed to be resistant based on the detection of inducible clindamycin resistance. * These isolates were used for whole-genome sequencing.

**Table 2:** Aminoglycoside susceptibility profiles of genetically related strains for which the presence of aminoglycoside-modifying enzymes (AMEs) does not explain the increase in MIC.

Strains	Patient	Visit	MLVA	*mecA*	MIC (µg/mL) ^a^		AMEs ^b^		
					GEN	TOB	AAC	ANT4	ANT9
CF1A-L	1	V1	A		0.25–0.5	0.5–1	-	-	-
CF54A-L	1	V3	A		2–4	2–4	-	-	-
CF54A-S	1	V4	A		>32	>32	-	-	-
CF2C-L	2	V1	D		2	2	-	-	-
CF2B-S	2	V1	D		4	4–8	-	-	-
CF4B-L	3	V1	A		1	1	-	-	-
CF4B-S	3	V1	A		4–8	4–8	-	-	-
CF28A-L	3	V2	G		0.5	0.5	-	-	-
CF63A-S	3	V3	G		4	>32	-	-	-
CF63A-L	3	V3	G		4–8	>32	-	-	-
CF5A-L	4	V1	H		0.25	0.5	-	-	-
CF5C-S	4	V1	H		8	4–8	-	-	-
CF7A-L	6	V1	K	+	0.5	>32	+	+	-
CF27A-L	6	V2	K	+	2–4	>32	+	+	-
CF48A-L	7	V3	K	+	1	>32	-	-	+
CF48A-S	7	V3	K	+	4	>32	-	-	+
CF51A-L	8	V2	P		2–4	2–4	-	-	-
CF51B-S	8	V2	P		8–16	4–8	-	-	-
CF10B-L	9	V1	R		0.5–1	0.5–1	-	-	-
CF10A-S	9	V1	R		>32	>32	-	-	-

^a^ Gentamicin (GEN), tobramycin (TOB). ^b^ Aminoglycoside-modifying enzymes (AMEs): *aac(6′)-aph(2′′)* [AAC], *ant(4′)-1* [ANT4], *ant(9)-1a* [ANT9].

## Data Availability

Genome sequences are available on NCBI (SRA data: PRJNA973120 Temporary Submission ID: SUB13343251) and MICs are available in Table S1.

## Supplementary Materials

The following supporting information can be downloaded at: https://www.mdpi.com/article/10.3390/antibiotics12061069/s1, Table S1: Clonal diversity and antibiotic minimal inhibitory concentrations (MICs) for 77 *S. aureus* isolates from 18 adult CF patients. Table S2: Possible SCV-inducting high-impact SNP mutations found in isolates CF5C-S and CF48B-S of unknown auxotrophy. Table S3: High- and moderate-impact SNP mutations found in CF28A-L, CF63A-L and CF63A-S isolates showing changes in antibiotic susceptibility over time. Figure S1: Auxotrophy complementation of a SCV by its co-isolated prototypical isolate.
