# Supplementary material for: Staphylococcus aureus Small-Colony Variants from Airways of Adult Cystic Fibrosis Patients as Precursors of Adaptive Antibiotic-Resistant Mutations
Source: Antibiotics (Basel). 2023 Jun 17;12(6):1069. doi: 10.3390/antibiotics12061069 (PMC10294822; doi:10.3390/antibiotics12061069)
Supplement: Supplementary file 1 [file antibiotics-12-01069-s001.zip › Figure S1_Auxotrophy complementation.pdf]

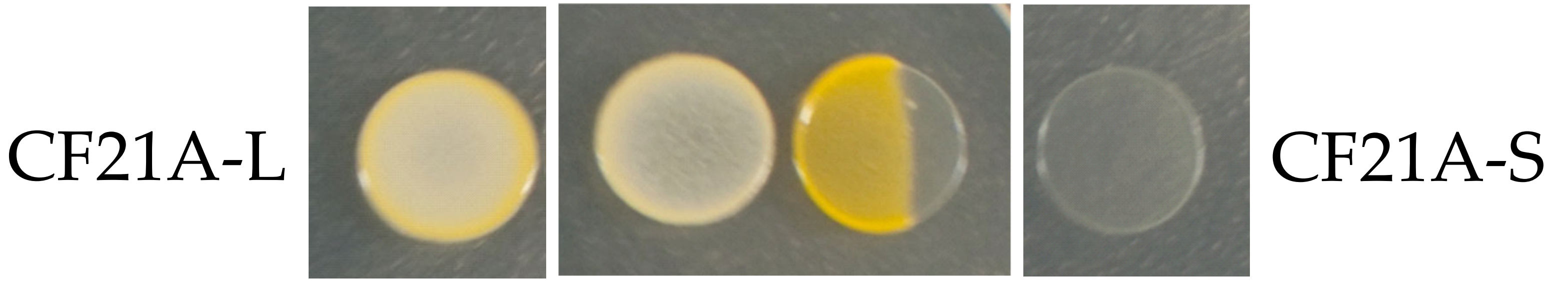

Figure S1. Auxotrophy complementation of a SCV by its co-isolated prototypical isolate. The prototypical isolate CF21A-L from patient 7 complements for growth and pigmentation the genetically-related SCV CF21A-S when bacteria are grown side by side on a TSA plate.
