# Supplementary material for: Staphylococcus aureus Small-Colony Variants from Airways of Adult Cystic Fibrosis Patients as Precursors of Adaptive Antibiotic-Resistant Mutations
Source: Antibiotics (Basel). 2023 Jun 17;12(6):1069. doi: 10.3390/antibiotics12061069 (PMC10294822; doi:10.3390/antibiotics12061069)
Supplement: Supplementary file 1 [file antibiotics-12-01069-s001.zip › TABLE S1_Detailed ntibiotic MIC.pdf]

TABLE S1. Clonal diversity and antibiotic minimal inhibitory concentrations (MICs) for 77 *S. aureus* isolates from 18 adult CF patients.

Patient	Visit	First isolate	Variant	MLVA	Agr	Minimal inhibitory concentration (μg/ml)										Aux
						OXA[a]	TOB	GEN	TMP-SXT[b]	CIP	ERY	CLI	TET	VAN	RIF	
		ATCC 29213				0.25	1	1	0.5/9.5	0.5	0.25-0.5	0.12	0.12-0.5	1-2	0.015	
1	V1	CF1A-L		A	2	0.5	0.5-1	0.25-0.5	0.06/1.2	0.12-0.25	0.06-0.12	0.12	0.06-0.12	1-2	0.008-0.02	
1	V1		CF1C-S	A	2	0.25-0.5	4-8 (I)	4-8 (I)	0.5/9.5	0.25	0.12	0.12	0.12	2	0.015	H
1	V1		CF1D-S	A	2	NT	8 (I)	2	NT	0.12	0.06-0.12	>32 (R)	0.06-0.25	2	0.003-0.025	U
1	V2		CF37B-S	A	2	1	4-8 (I)	4-8 (I)	1/19	>64	>64 (R)	NT	0.06-0.12	2	0.008-0.015	U
1	V3		CF54A-L	A	2	0.25-0.5	2-4	2-4	1/19	0.5	0.25-0.5	NT	0.12-0.25	1	0.015-0.03	
1	V4		CF54A-S	A	2	0.5	>32 (R)	>32 (R)	1/19-2/38	0.5	0.25	NT	0.12-0.25	1	0.015-0.03	U
1	V2	CF37A-L		B	2	>128 (R)	2-4	1-2	0.5/9.5-1/19	>64	>64 (R)	NT	0.12-0.25	2	0.015-0.03	
1	V2		CF37A-S	B	2	>128 (R)	1	0.5-1	1/19	>64	>64 (R)	NT	0.12-0.5	2	0.015-0.03	U
2	V1	CF2A-L		C	1	0.5	0.25-0.5	0.25-1	0.06/1.2	0.25-0.5	0.25-0.5	0.12	0.5-2	0.5-1	0.025-0.06	
2	V3		CF62B-L	C	1	1	2-4	1-4	1/19	0.5-1	0.25	NT	1-2	2	0.015	
2	V1	CF2C-L		D	3	0.5	2	2	0.03/0.6	0.12	0.12	0.12	0.25	1	0.004	
2	V1		CF2B-S	D	3	0.5	4-8 (I)	4	0.12/2.4	0.12-0.25	0.06-0.25	0.12	0.06-0.12	1-2	0.015-0.06	H
2	V3		CF62A-L	D	3	1	2-8	4	1/19	0.5	0.25	NT	0.25	2-4	0.015	
2	V2	CF34A-L		E	1	0.5	2	0.5-2	0.06/1.2	0.25-0.5	0.12-0.5	NT	0.03-1	2	0.015-0.03	
2	V2	CF34B-L		F	3	1	1	0.5-1	0.06/1.2	0.5	0.25	NT	0.06-0.12	1-2	0.004-0.008	
3	V1	CF4B-L		A	2	0.5-1	1	1	0.06/1.2	0.5	0.25-0.5	0.12	0.12-0.5	1-2	0.015	
3	V1		CF4B-S	A	2	0.5	4-8 (I)	1-8 (I)	0.06/1.2	0.5	0.06-0.12	<0.06	0.03-0.06	2	0.03-0.05	M
3	V2		CF28B-L	A	2	1	1	1	0.06/1.2	0.25-0.5	0.25-0.5	NT	0.06	2	0.008-0.015	
3	V2	CF28A-L		G	2	2	0.5	0.5	0.03/0.6	0.25	>64 (R)	NT	0.12	0.5-1	0.008-0.015	
3	V3		CF63A-S	G	2	1	>32 (R)	4	1/19	0.25-1	>64 (R)	NT	0.12-0.25	1	0.015	U
3	V3		CF63A-L	G	2	1	>32 (R)	8 (I)	1/19	16 (R)	>64 (R)	NT	0.12-0.25	1-2	0.015-0.03	
4	V1	CF5A-L		H	3	0.25	0.5	0.25	0.03/0.6	1-2	0.03-0.12	0.12-0.25	0.03-0.06	1	0.03-0.05	
4	V1		CF5C-S	H	3	NT	4-8 (I)	8 (I)	0.06/1.2	0.5-1	0.062	0.12	0.03-1	1	0.002-0.015	U
4	V1	CF5E-S		I	3	0.12	8 (I)	8 (I)	0.03/0.6	1	0.062	0.12	0.06	2	0.002	H+M
5	V1	CF6B-L		J	3	0.5	>32 (R)	>32 (R)	0.12/2.4	2	0.25	0.06	0.06-0.12	1	0.008-0.015	
5	V1		CF6A-S	J	3	0.5	>32 (R)	>32 (R)	8/152 (R)	0.25-0.5	0.5	0.12-0.25	0.12-0.25	2	0.015-0.03	T
5	V1		CF6C-S	J	3	0.5	>32 (R)	>32 (R)	0.25/4.8	0.25	0.25	0.12	0.06-0.12	1	0.008-0.015	H+M
5	V2		CF39A-L	J	3	0.5	>32 (R)	>32 (R)	8/152 (R)	2	0.5-1	NT	0.25	1	0.03	
5	V2		CF39A-S	J	3	0.25	>32 (R)	>32 (R)	8/152 (R)	2	0.5-1	NT	0.25	1	0.015-0.03	U
6	V1	CF7A-L		K	2	>128 (R)	>32 (R)	0.5	0.06/1.2	>64 (R)	>64 (R)	>32 (R)	0.06-0.12	1-2	0.015-0.03	
6	V1		CF7D-L	K	2	0.25	0.5	0.5	0.03/0.6	>64 (R)	>64 (R)	>32 (R)	0.06-0.12	1	0.015-0.03	
6	V2		CF27A-L	K	2	>128 (R)	>32 (R)	2-4	0.5/9.5-1/19	>64 (R)	>64 (R)	NT	0.12-0.25	2	0.015-0.06	
6	V3	CF49A-L		L	3	0.25	4	2-4	0.5/9.5-1/19	1	0.25-0.5	NT	0.12-0.25	2-4	0.03	
6	V3	CF49A-S		M	3	0.25	8 (I)	4	0.06/1.2	0.12-0.25	0.25-0.5	NT	0.03- 0.06	2	0.03	H
6	V3		CF49B-S	M	3	0.25-0.5	8 (I)	4	1/19	0.5-1	0.25-0.5	NT	0.25-1	2-4	0.015-0.12	U
7	V1	CF8A-L		N	3	0.25	0.5	0.5	0.06/1.2	1	1 (I)	0.12	0.5	2	0.008	
7	V2		CF21A-L	N	3	0.25	1	1-2	1/19	0.25-0.5	0.25	NT	0.06-0.5	1	0.008-0.03	
7	V2		CF21A-S	N	3	0.125	4	2	1/19	0.25	0.125	NT	0.5	1-2	0.015	M
7	V1	CF8C-L		O	3	0.5	1-2	1-2	0.06/1.2	0.25-0.5	0.25	0.12-0.25	0.12-0.25	2-4	0.008-0.06	
7	V1		CF21D-L	O	3	0.5-1	2	0.5-1	2/38	4	0.25	NT	0.12-0.25	1-2	0.015-0.12	
7	V1	CF8D-L		P	3	0.12	4	4	0.06/1.2	0.25	0.25	0.12-0.25	0.12	2-4	0.004	
7	V3	CF48A-L		K	2	>128 (R)	>32 (R)	1	0.25/4.8	>64 (R)	>64 (R)	NT	0.12-0.25	2-4	0.03	
7	V3		CF48A-S	K	2	64 (R)	>32 (R)	2-4	0.5/9.5	>64 (R)	>64 (R)	NT	0.12-0.25	1-2	0.008-0.06	U
7	V3		CF48B-S	K	2	0.25	>32 (R)	8 (I)	1/19	>64 (R)	>64 (R)	NT	0.12-0.25	4 (I)	0.015-0.03	U

Patient	Visit	First isolate	Variant	MLVA	Agr	Minimal inhibitory concentration (μg/ml)										Aux
						OXA[a]	TOB	GEN	TMP-SXT[b]	CIP	ERY	CLI	TET	VAN	RIF	
		ATCC 29213				0.25	1	1	0.5/9.5	0.5	0.25-0.5	0.12	0.12-0.5	1-2	0.015	
8	V1	CF9A-L		K	2	>128 (R)	>32 (R)	0.5-2	0.06/1.2	>64 (R)	>64 (R)	0.12 (R)[c]	0.06-0.25	1	0.03-0.06	
8	V1		CF9F-L	K	2	0.5	>32 (R)	>32 (R)	0.06/1.2	>64 (R)	>64 (R)	2 (I)	0.25-0.5	0.5-2	0.015-0.06	
8	V2	CF51A-L		p	2	0.25	2-8	2-4	0.5/9.5	>64 (R)	>64 (R)	NT	1	2	0.015-0.03	
8	V2		CF51B-S	p	2	0.25	8-16 (I)	4-8 (I)	0.5/9.5-1/19	>64 (R)	>64 (R)	NT	0.5-1	4 (I)	0.015-0.03	U
8	V3		CF75B-L	P	2	0.25	1-4	1-2	0.5/9.5	>64 (R)	>64 (R)	NT	0.5-1	1-2	0.03-0.06	
8	V3	CF75A-L		Q	1	1	2	1	0.5/9.5	>64 (R)	0.5	NT	0.25-0.5	2-4	0.015	
9	V1	CF10B-L		R	1	1	0.5-1	0.5-1	0.12/2.4	0.12-0.5	>64 (R)	16-32 (R)	0.12-0.25	1	0.008-0.03	
9	V1		CF10A-S	R	1	0.25	>32 (R)	>32 (R)	0.06/1.2	0.25	>64 (R)	16-32 (R)	0.06	1-2	0.008	H+M
10	V1	CF18A-L		S	2	0.5	1-4	0.5-4	0.03/0.6	0.12-0.5	>64 (R)	0.12 (R)[c]	0.12-0.25	1	0.008-0.015	
10	V2		CF33A-L	S	2	1	1	1-4	0.5/9.5-1/19	1-2	>64 (R)	NT	0.12-0.25	2	0.008-0.015	
10	V3		CF78A-L	S	2	0.25	4-8 (I)	2-4	0.5/9.5-1/19	0.25-0.5	0.25-0.5	NT	0.25	1	0.015	
10	V3		CF78A-S	S	2	0.25	4-16 (I)	1-2	0.5/9.5	0.25-0.5	0.25-0.5	NT	0.12	1	0.015	M
10	V3		CF78B-S	S	2	0.12-0.25	8-16 (I)	4	1/19	0.12-0.25	0.25	NT	0.12	1	0.008-0.015	H
10	V1	CF18E-L		T	1	1	1	0.5-1	0.25/4.8	0.25	0.25	<0.06	0.12-0.25	1-4	0.004-0.015	
11	V1	CF19A-L		T	1	0.5-1	1	0.5-1	0.03/0.6	4-8 (R)	0.12-0.25	0.12	0.12-0.25	1-2	0.008-0.015	
11	V1	CF19B-L		U	1	0.5	2	2	0.06/1.2	4 (R)	0.12	0.12	0.12-0.25	2-4	0.008	
11	V1	CF19A-S		V	1	1	>32 (R)	>32 (R)	0.25/4.8	1	0.25	0.12	0.06-0.25	1-2	0.008-0.015	U
11	V2	CF41A-L		W	1	0.5	2-4	2	0.06/1.2	16 (R)	64 (R)	NT	0.25	2-4	0.03-0.06	
11	V2		CF41A-S	W	1	0.5	1-8 (I)	2-4	0.5/9.5-1/19	16 (R)	0.5	NT	0.12	2	0.015-0.03	U
11	V3	CF58A-L		X	1	0.25-1	>32 (R)	>32 (R)	>8/152 (R)	1	0.25	NT	0.12	2	0.015	
11	V3	CF58B-L		Y	1	0.5-1	8 (I)	4	0.5/9.5	32 (R)	0.25-2	NT	0.25-0.5	2-4	0.03	
12	V1	CF22A-L		Z	1	0.5-1	0.5-4	0.5	0.12/2.4	0.25-0.5	0.25	0.06	0.12	1-2	0.015-0.06	
13	V1	CF29A-L		AA	1	0.5	8 (I)	4-8 (I)	0.06/1.2	0.25	0.25	>32 (R)	0.12-0.25	1-4	0.008-0.015	
14	V1	CF35A-L		K	2	>128 (R)	1	1	0.06/1.2	>64 (R)	>64 (R)	0.12 (R)[c]	0.12	2-4	0.015-0.03	
14	V2		CF77A-L	K	2	>128 (R)	1-2	1-2	1/19	>64 (R)	>64 (R)	NT	0.25-1	1-2	0.03-0.06	
15	V1	CF43A-L		BB	3	2	0.5-1	1	0.12/2.4	8 (R)	>64 (R)	>32 (R)	0.12	1-2	0.004-0.008	
15	V1	CF43B-L		CC	3	1	2	1-2	0.06/1.2	4-16 (R)	>64 (R)	>32 (R)	0.25	2	0.008	
16	V1	CF50A-L		K	2	>128 (R)	>32 (R)	1	0.5/9.5	>64 (R)	>64 (R)	0.12	0.25-0.5	2	0.015-0.03	
16	V1		CF50A-S	K	2	>128 (R)	>32 (R)	4-8 (I)	1/19	>64 (R)	>64 (R)	0.12	0.25-0.5	2-4	0.03	H
16	V2	CF111A-L		DD	2	64 (R)	>32 (R)	1-2	0.25/4.8	>64 (R)	>64 (R)	NT	0.25	1-2	0.015	
17	V2	CF86A-S		EE	2	0.25	2-4	0.5-1	1/19	0.5	0.25	NT	>16 (R)	1-2	0.015-0.03	H
18	V1	CF81A-L		FF	3	2	0.5	0.5	0.12/2.4	0.25	0.25-0.5	0.12-0.25	0.12-1	1-2	0.015	
18	V2	CF91A-L		GG	1	0.5	1	0.5	0.12/2.4	0.25	0.12-0.25	NT	0.12	4 (I)	0.015-0.03	
total: 77 isolates			MIC range			0.12-128	0.25-32	0.25-32	0.03/0.6- 8/152	0.12-64	0.03-64	0.06-32	0.03-16	0.5-4.0	0.002-0.12	
			MIC50			0.5	8	2	0.25/4.75	1	0.5	0.12	0.25	2	0.03	
			MIC90			>128	>32	>32	1/19	>64	>64	>32	1	4	0.06	
			% resistant (R/tested)			16	27	13	5	36	38	30	1	0	0	
