# Supplementary material for: Staphylococcus aureus Small-Colony Variants from Airways of Adult Cystic Fibrosis Patients as Precursors of Adaptive Antibiotic-Resistant Mutations
Source: Antibiotics (Basel). 2023 Jun 17;12(6):1069. doi: 10.3390/antibiotics12061069 (PMC10294822; doi:10.3390/antibiotics12061069)
Supplement: Supplementary file 1 [file antibiotics-12-01069-s001.zip › TableS2_reviewfinal.pdf]

Table S2. Possible SCV-inducting high-impact SNP mutations found in isolates CF5C-S and CF48B-S of unknown auxotrophy.

Isolate	Patient/Visit	Gene altered *vs*. related prototypical isolate	Mutation found
CF5C-S	4/V1	NWMN_2298 *cysG*; uroporphyrin-III C-methyl transferase<br>Pathway altered: Porphyrin metabolism	16>17insGGTT;<br>Tyr6fs
CF48B-S	7/V3	NWMN_0911 *menA*; 1,4-dihydroxy-2-naphthoate octaprenyltransferase<br>Pathway altered: Menaquinone biosynthesis	133G>T;<br>Glu45*
