# Supplementary material for: Staphylococcus aureus Small-Colony Variants from Airways of Adult Cystic Fibrosis Patients as Precursors of Adaptive Antibiotic-Resistant Mutations
Source: Antibiotics (Basel). 2023 Jun 17;12(6):1069. doi: 10.3390/antibiotics12061069 (PMC10294822; doi:10.3390/antibiotics12061069)
Supplement: Supplementary file 1 [file antibiotics-12-01069-s001.zip › TableS3_review.pdf]

Table S3. High- and moderate-impact SNP mutations found in CF28A-L, CF63A-L and CF63A-S isolates showing changes in antibiotic susceptibility over time.

Isolate	Patient 3 visit	Antibiotic resistance [a]	Gene altered	Mutation found
CF28A-L	V2	None	NWMN_0456 *ksgA*; dimethyladenosine transferase	Frameshift removing the initiation codon
CF63A-S<br>CF63A-L	V3<br>V3	TOB (R), ERY (R)<br>TOB (R), GEN (I), ERY (R), CIP (R)	NWMN_0456 *ksgA*; dimethyladenosine transferase	Frameshift removing the initiation codon
			NWMN_2096 *lacD*; tagatose 1,6-diphosphate aldolase	730C>T; Pro244Ser

[a] TOB, tobramycin; GEN, gentamicin; ERY, erythromycin; CIP, ciprofloxacin; R, resistant; I, intermediate. See precise minimal inhibitory concentrations in Table S1.
